# Antimicrobial peptides as drugs with double response against *Mycobacterium tuberculosis* coinfections in lung cancer

**DOI:** 10.3389/fmicb.2023.1183247

**Published:** 2023-06-02

**Authors:** Giulia Polinário, Laura Maria Duran Gleriani Primo, Maiara Alane Baraldi Cerquetani Rosa, Freddy Humberto Marin Dett, Paula Aboud Barbugli, Cesar Augusto Roque-Borda, Fernando Rogério Pavan

**Affiliations:** ^1^School of Pharmaceutical Sciences, São Paulo State University (UNESP), Araraquara, São Paulo, Brazil; ^2^Vicerrectorado de Investigación, Universidad Católica de Santa María (UCSM), Arequipa, Peru

**Keywords:** *Mycobacterium tuberculosis*, lung cancer, peptides, multifunctional peptides, treatment

## Abstract

Tuberculosis and lung cancer are, in many cases, correlated diseases that can be confused because they have similar symptoms. Many meta-analyses have proven that there is a greater chance of developing lung cancer in patients who have active pulmonary tuberculosis. It is, therefore, important to monitor the patient for a long time after recovery and search for combined therapies that can treat both diseases, as well as face the great problem of drug resistance. Peptides are molecules derived from the breakdown of proteins, and the membranolytic class is already being studied. It has been proposed that these molecules destabilize cellular homeostasis, performing a dual antimicrobial and anticancer function and offering several possibilities of adaptation for adequate delivery and action. In this review, we focus on two important reason for the use of multifunctional peptides or peptides, namely the double activity and no harmful effects on humans. We review some of the main antimicrobial and anti-inflammatory bioactive peptides and highlight four that have anti-tuberculosis and anti-cancer activity, which may contribute to obtaining drugs with this dual functionality.

## Graphical Abstract

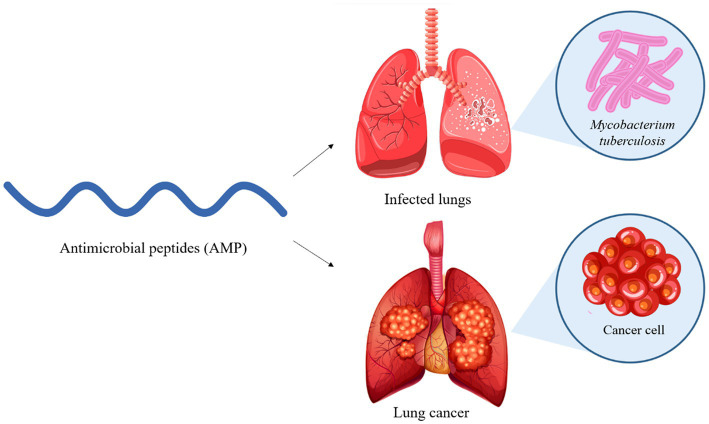

## 1. Introduction

### 1.1. Tuberculosis

Tuberculosis, a disease caused by *Mycobacterium tuberculosis* (Mtb), usually affects the lungs but can also affect other sites (extrapulmonary tuberculosis; Gradmann, 2006). First contact occurs when the bacillus is inhaled and it reaches the lungs. It may then be eliminated by the immune system, contained in a state of latency, or initiate an active infection. The outcome differs for each individual, depending on the immune status of the host (Ankrah et al., 2018). Common symptoms are coughing with sputum and sometimes blood, chest pain, weakness, weight loss, fever, and night sweats. These symptoms are often mild for many months, leading to delays in treatment and increasing the risk of spreading the infection to others (Bloom and Murray, 1992).

Until the COVID-19 pandemic, tuberculosis was considered the leading cause of death from a single infectious agent. According to the report by the World Health Organization, 5.8 million people were diagnosed with the disease in 2020, a lower number than in 2019, probably affected by the pandemic, but the figure rose again in 2021 to 6.4 million. With the drop in the number of diagnoses and, consequently, treatment, the number of deaths increased, to officially 1.4 million deaths in 2021. These data are a consequence of several factors associated with the COVID-19 pandemic: the health system was less capable of receiving tuberculosis cases, people had limited mobility and the symptoms of the disease and COVID were similar. It is believed that 10.6 million people contracted tuberculosis in 2021, a significantly higher number than those diagnosed. Most of these cases occurred in Southeast Asia (45%), Africa (23%) and the Western Pacific (18%). Men were the most affected by the disease (56.5% of total cases), followed by women (32.5%) and children (11%; WHO, 2022).

### 1.2. Lung cancer

Lung cancer (LC) is another serious disease that affected 2.2 million people in 2020 (11.4% of all cancers), killing 1.7 million, about 18% of all cancer deaths in the world (Globocan, 2020). Currently, this type of cancer has a poor prognosis and adverse clinical outcomes, mainly because of its late diagnosis, ineffective treatment and tumor cell resistance. There are about 2.09 million cases and 1.76 million deaths every year (Arrieta et al., 2022). It is the second most common diagnosis in men and women, after prostate and breast cancer. Most LC cases occur at a mean age of 70 years and men are more affected than women (Chaitanya Thandra et al., 2021). Table 1 lists some microorganisms present in the tumor microenvironment, as well as their possible interactions with cancer cells.

**Table 1:** Microorganisms present in LC patients (oncobiome of lung cancer patients).

Microorganism	Interaction	References
*Mycobacterium tuberculosis*	Suspected initiation and proliferation, colonization	Pilaniya et al. (2016), Cameron et al. (2017), Mao et al. (2018), Poore et al. (2020)
*Staphylococcus genera. Species like epidermis and aureus*	Colonization	Cameron et al. (2017), Poore et al. (2020)
*Propionibacterium acnes*	Colonization	Poore et al. (2020)
*Ralstonia* spp.	Colonization	Poore et al. (2020)
*Pseudomonas*	Suspected initiation and proliferation, colonization	Poore et al. (2020)
*Acinetobacter*	Colonization	Cameron et al. (2017), Poore et al. (2020)
*Haemophilus influenzae*	Colonization	Laroumagne et al. (2011), Mao et al. (2018)
*Enterobacter spp.*	Colonization	Laroumagne et al. (2011), Mao et al. (2018)
*Escherichia coli*	Colonization	Laroumagne et al. (2011), Mao et al. (2018)
*Granulicatella*	Colonization	Yan et al. (2015), Cameron et al. (2017), Mao et al. (2018)
*Abriotrophia*	Colonization	Yan et al. (2015), Mao et al. (2018)
*Streptococcus*	Colonization	Yan et al. (2015), Cameron et al. (2017), Mao et al. (2018), Tsay et al. (2018)
*Capnocytophaga*	Colonization	Hosgood et al. (2014), Yan et al. (2015), Mao et al. (2018)
*Selenomonas*	Colonization	Yan et al. (2015), Mao et al. (2018)
*Veillonella*	Colonization	Hosgood et al. (2014), Yan et al. (2015), Mao et al. (2018), Tsay et al. (2018), Zhao et al. (2021)
*Neisseria*	Colonization	Yan et al. (2015), Cameron et al. (2017), Mao et al. (2018)
*Legionella*	Metastasis	Yu et al. (2016), Mao et al. (2018)
*Thermus*	Colonization	Yu et al. (2016), Mao et al. (2018)
*Enterococcus*	Colonization	Cameron et al. (2017), Zhuang et al. (2019), Zhao et al. (2021)
*Akkermansia muciniphila*	Suspected initiation	Zhao et al. (2021)
*Rhodococcus erythropolis*	Colonization	Cameron et al. (2017)
*Stenotrophomonas maltophilia*	Colonization	Cameron et al. (2017)
*Acidovorax*	Suspected initiation and colonization	Greathouse et al. (2018)

To understand the entire pathophysiology of LC, one must understand the contributions of the tumor microenvironment (TME) and the host immune responses, which are interconnected with each step of the tumorigenesis of each cancer subtype (Salehi-Rad et al., 2020). The TME is the environment where the tumor is located and is formed by various types of cells, like immune and stromal cells. Additionally, the TME is composed of extracellular matrix molecules and a variety of cytokines produced by the tumoral cells (Altorki et al., 2019; Wu et al., 2021). This microenvironment can define the cells present within the tumor, as well as the cytokines and the interactions that may occur. For example, macrophages and neutrophils are involved in the mechanism of immune escape and lung cancer development. These cells produce a proinflammatory background that strongly affects the carcinogenesis and the immune-response efficiency (Altorki et al., 2019; Madeddu et al., 2022). The extracellular matrix is composed of collagens, proteoglycans and glycosaminoglycans, and it mediates the interaction between the cells in the TME. This interaction can promote carcinogenesis (Altorki et al., 2019). The TME is also important for the evasion of immune recognition. Lung cancers can alter the composition of TME to establish an immunosuppressive environment, by increasing the abundance of inhibitory molecules, such as TGT-β, IL-6 and PGE2 (Salehi-Rad et al., 2020). The genomic profile is defined by the progressive accumulation of mutations in oncogenes and tumor suppressor genes, from dysplasia and pre-neoplasia to metastatic cancer (Hanahan and Weinberg, 2011). Intrinsic genomic alterations within premalignant or neoplastic cells can reprogram the composition of the TME to facilitate carcinogenesis. Still, chronic inflammation caused by extrinsic factors, such as unresolved infections, can induce the lung TME to develop cancer progression (Dubinett, 2015). Detailed genomic profiling of LC has revealed significant heterogeneity among patients, and this has important clinical implications for the evolution of the cancer genome and acquired resistance to therapy (Salehi-Rad et al., 2020).

There are two main types of LC, called ‘small cell’ (approximately 15%) and ‘non-small cell’ (the remaining 85%). The most frequently found, non-small cell (NSCLC), is most associated with smoking but is also found in people who have never smoked, with a slightly different genomic landscape. This LC type includes subtypes with treatment and prognoses that are often similar, the main subtypes being adenocarcinoma, squamous cell carcinoma and large cell carcinoma. The small cell type (SCLC), in most cases, is a high-grade neuroendocrine tumor. This type is characterized by fast growth and metastasis, as compared with NSCLC type, and a poor clinical outcome in general (Gazdar et al., 2017; American Cancer Society, 2019). As it is a highly heterogeneous disease, molecular characterization is claimed to improve the understanding of tumor pathogenesis and thus define a personalized treatment plan (De Castro et al., 2019).

Some factors facilitate the development of this carcinoma, such as age, tobacco smoking and tuberculosis (Chaitanya Thandra et al., 2021). Several types of studies have shown that there is a positive correlation between the incidence of tuberculosis and LC, and the simultaneous occurrence of these features has increased. Tuberculosis patients have a 50% increased chance of developing LC. Those with a history of tuberculosis for more than 20 years are 2.5 times more likely to develop this disease (Sun et al., 2022). Therefore, these patients must be monitored to prevent the development of this type of cancer, since the prognosis of the disease can also be affected (Arrieta et al., 2022).

### 1.3. Tuberculosis and lung cancer correlation

Many studies have demonstrated the relationship between chronic inflammation and cancer development. In 1863, Rudolf Virchow first identified the correlation between inflammation and cancer when he noticed that there were leukocytes in neoplastic tissues (Balkwill and Mantovani, 2001). Experimental evidence has proved that lung carcinoma can be triggered by chronic Mtb infection (Nalbandian et al., 2009). Furthermore, the most recent meta-analysis we found demonstrates that pre-existing active pulmonary TB increases the relative risk of LC, mainly squamous cell carcinoma (3,570), followed by adenocarcinoma (2,605) and small cell carcinoma (2,118). It also exhibits an increased risk of 2,746 for other histological types of LC. The authors of this meta-analysis searched for articles and abstracts published from 1987 to 2021 in different databases, and they concluded that a patient with a history of active pulmonary TB should be followed up for a longer time after the cure of pulmonary TB than patients with no such history (Abdeahad et al., 2022). Another study also showed that the incidence of LC was higher in patients with tuberculosis, with a significant increase in mortality in TB cancer patients (Kiri et al., 2009; Liang et al., 2009). In this regard, it is important to highlight that TB treatment usually takes 6–9 months of combined drug therapy. During this period, TB infection causes severe lung inflammation, which could be related to a chronic inflammatory process that has been linked to several stages of carcinogenesis (Liu et al., 2020).

Although both diseases affect the same anatomical unit of the lung, some researchers do not believe that there is clear pathogenesis between them, but rather that tuberculosis is just another direct risk factor for LC, among other widely known factors (Qin et al., 2022). As both diseases affect the same organ, the symptoms are very similar, and although it is harmful for a cancer patient to receive tuberculosis treatment (or vice versa), it is even more dangerous for a patient with both diseases to receive treatment for only one of them, as the other disease worsens without treatment (Malik et al., 2022).

The immune system plays an important role in cancer prevention; however, cancer cells can still act by preventing or inhibiting antitumor responses. Innate and adaptive immunities work by eliminating or suppressing viral infections that can induce a tumor, preventing the maintenance of an environment that favors the emergence of a tumor or identifying tumor cells and eliminating them (Shroff et al., 2022).

### 1.4. Tuberculosis infection and lung cancer development

**Figure 1:**
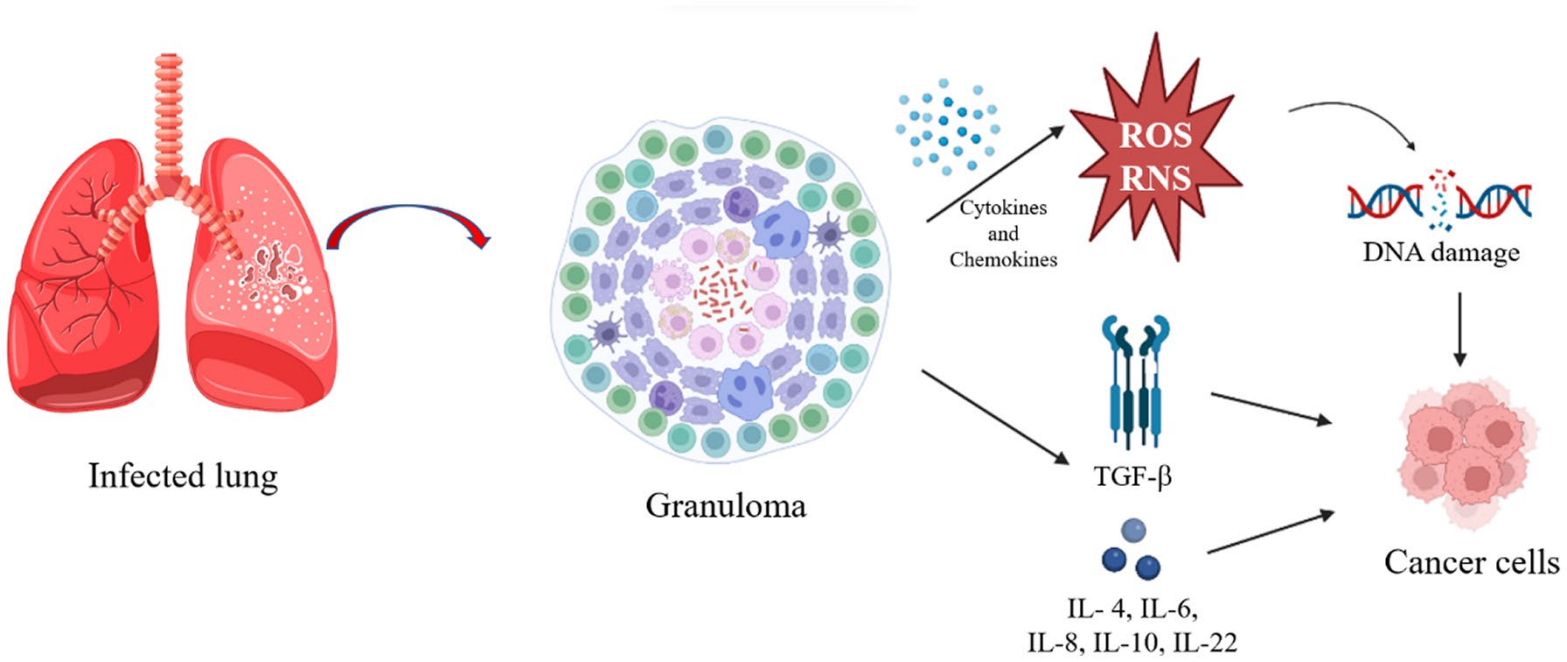
Pathway triggered after infection that can result in cancer cells. The figure was created by Biorender.com, partly generated using Servier Medical Art, provided by Servier, licensed under a Creative Commons Attribution 3.0 unported license.

Mtb is an intracellular pathogen able to infect human mononuclear phagocytes, spending most of its life cycle in macrophages (Vasava et al., 2017). In addition to other very important immune cells, in the initial phase of Mtb infection, the bacillus is phagocytosed by alveolar macrophages (AM). These cells are then activated and they adopt a pro-inflammatory phenotype, recruiting more immune cells to the site of infection, among other interstitial macrophages (IM), which decrease oxidative phosphorylation, the main way of obtaining energy from AM (Tukiman, 2022). This type of energy acquisition is mainly fueled by the oxidation of fatty acids, which are abundant in lung tissue, but this makes the environment even more conducive to infection by mycobacteria, which also use fatty acids as a carbon source (Sheedy and Divangahi, 2021). IM also increases aerobic glycolysis, as in the Warburg effect, providing biosynthetic precursors necessary for rapid cell growth and proliferation (Macintyre and Rathmell, 2013). The Warburg effect has been described to explain the origin and functioning of cancer cells, and it includes the generation of pro-inflammatory cytokines and antimicrobial molecules, in addition to reactive oxygen and nitrogen species (ROS and RNS; Shi et al., 2016a). This mechanism is part of the attempt to eliminate the infection, so if aerobic glycolysis is inhibited at this stage of the infection, the resulting immunological changes lead to an increase in Mtb survival, mainly due to the reduction in pro-inflammatory IL-1β levels (Gleeson et al., 2016). Inflammatory cells produce many cytokines and chemokines that form an attractive environment for tumor development, promoting the formation of new blood vessels from existing ones, in addition to facilitating genomic instability (Coussens and Werb, 2002). In chronic inflammation, by producing ROS and RNS, most phagocytic cells induce DNA damage in proliferating cells due to the consequent production of peroxynitrite (Maeda and Akaike, 1998). If DNA damage occurs continuously and repeatedly, it may result in permanent genomic alterations, such as point mutations, deletions or rearrangements (Coussens and Werb, 2002; Figure 1).

In the early stage of this infection, activation of the immune response with type T helper cells (Th1) and the production of IFN-γ and TNF-α are the most prominent protective mechanisms for intracellular mycobacterial killing. With the progression of the infection and the interaction between mycobacteria and immune cells, the formation of granulomas occurs. These granulomas are characterized by an extremely hypoxic and inflammatory internal region, maintained by glycolysis to kill the bacilli, and a less inflammatory peripheral region, consequently favoring the survival of the bacillus (Tukiman, 2022). Granulomas can lead to caseous lesions and cavity formation, in the latter case being extremely infectious as it is the consequence of lung tissue destruction with the formation of macroscopic open spaces that contain numerous bacilli and connect with the large airways, facilitating efficient expectoration of the bacteria (Younga and Verreck, 2012). The granuloma is characterized by glycolysis in the central region and oxidative phosphorylation in the peripheral region. These granulomas normally contain live mycobacteria, myeloid and lymphoid cells (Nalbandian et al., 2009). However, the Warburg effect was found to be reduced in the nucleus of the granuloma, suggesting that the bacillus can modulate the host’s defense mechanism to survive (Shi et al., 2016a,b). In line with this finding, Shi et al. (2016a) suggested the search for therapeutic compounds that have the potential to enhance the Warburg effect on infected macrophages to combat Mtb (Shi et al., 2016a).

Finally, LC originating from the dysregulated chronic inflammation can also foster abundant expression of growth factors and cytokines, such as transforming growth factor beta (TGF-b) and interleukin (IL)-1b, IL-4, IL-6, IL-8, IL-10, and IL-22, which activate multiple tumorigenic pathways, such as cyclooxygenase 2 (COX-2) and nuclear factor kappa B subunit (NF-kB) to promote tumorigenesis (Dubinett, 2015). More importantly, TB also causes extensive pulmonary fibrosis, which is associated with the production of TGF-Β, IL-4 and IL-13 (Heitmann et al., 2014; George et al., 2015). Conversely, LC can decrease local immunity and reactivate a latent infection, or even increase the chances of an exogenous infection, so it is worth paying close attention to these diseases (Dobler et al., 2017).

An important epithelial growth factor produced by the mycobacteria in the early stages of tumor formation is epiregulin. In inflammatory macrophages, its production can be beneficial, as it helps to repair tissue damage; however, with the presence of persistent live pathogens, such as Mtb, this adaptive response is directed against the host, providing in this case, a potent growth factor for pre-malignant cells (Nalbandian et al., 2009). High levels of expression of this gene have been found in several cancer cell lines (Baba et al., 2000).

The effect of pulmonary TB on the epidermal growth factor receptor (EGFR) in patients with lung adenocarcinoma (LAC) was investigated in 2019, and a significantly higher mutation frequency was found than in patients without tuberculosis (Hwang et al., 2019). Mutations in this gene, which originally encodes proteins for cell proliferation and survival, are the most frequent in LAC (Jett and Carr, 2013), and TB-induced overexpression of epiregulin conferred invasive properties on cancer cells (Zhang et al., 2008).

Most lung damage resulting from an Mtb infection is the result of an intense inflammatory immune response. It has been proposed that this damage has different levels of severity depending on the variability of the genes that encode or regulate the host’s immune responses, but this hypothesis has not yet been proven (Ravimohan et al., 2018). In 2009, an Mtb genetic locus that specifically controls tissue damage and progression of pulmonary tuberculosis, *sst1*, was identified as an important genetic modifier that could lead to lung tumor formation (Nalbandian et al., 2009). Chai et al. (2020) demonstrated that TB and LAC share 65 similar signature genes, but emphasized the *MK167* gene, which encodes Ki-67, a protein expressed exclusively in proliferating cancer cells (Chai et al., 2020). The *MK167* gene was equally increased in TB and LAC groups, and it is an important mediator for Mtb-induced proliferation, migration and evasion of tumor cells. It was also discovered that this gene is regulated by an effector protein that can promote tumor development by regulating cell proliferation and migration when it enters the host cell nucleus (Wang et al., 2017). Chai et al. (2020) further suggested that the mechanism that the mycobacteria use to spread more efficiently in LC patients is through infected tumor cells, as they obtained positive mobility results in migration and invasion assays (Chai et al., 2020).

Cao et al. (2019) hypothesized that Mtb infection mediated immune response and facilitated tumor metastasis through the PD-1/PD-L1 signaling pathway (Cao et al., 2019). Mtb also reprograms macrophages during granuloma formation by inducing expression of E-cadherin into epithelioid morphology, a process analogous to epithelial-to-mesenchymal transition (EMT; Cronan et al., 2016; Ahmad et al., 2022). Further, Mtb upregulates transcription factors that induce EMT, a hallmark of carcinogenesis and metastatic progression (Hofman and Vouret-Craviari, 2012; Gupta et al., 2016). Some important up-regulated genes, such as *MYBL2, BRCA-1, UBE2C, CHEK-1, CDN2A* and *PCNA*, are common in LC and TB, mostly associated with cell cycle, checkpoints and apoptosis (Cao et al., 2019).

### 1.5. Membranolytic peptides

Peptides are molecules derived from proteins that can be obtained by hydrolysis, like bioactive peptides, or isolated from animal protein fluids (constitutive or induced) as a natural defense mechanism (Silveira et al., 2021). Membranolytic peptides can be responsible for destabilizing the cellular homeostasis of microorganisms, such as antimicrobial peptides (AMPs), and tumor cells, such as anticancer peptides (ACPs). The history of peptides began in 1922 when lysozyme was discovered (Fleming, 1922). Through proteomic characterization, lysozyme turned out to possess various AMPs with potential bactericidal activity (Ibrahim et al., 2011). AMPs are broad-spectrum biomacromolecules that, for the most part, are capable of interacting with various receptors on the cell membrane or wall, causing intra-and extracellular imbalance and later leading to death.

This type of peptide generally has less than 100-mers and is characterized by rapidly interacting with the pathogenic agent at low concentrations, decreasing the rate of induction or generation of resistance. Roque-Borda et al. (2021a) reported that the use of the antimicrobial peptide Ctx(Ile^21^)-Ha has great activity against *Pseudomonas aeurigonosa* and *Acinetobacter baumannii*, two of the deadliest bacteria in the world according to the WHO priority list (Roque-Borda et al., 2021b). It is known that these two bacteria can be more dangerous and deadly when they have genes for resistance to carbapenems since these drugs are used as the last lines of treatment. Da Costa de Souza et al. (2022) showed that AMPs are capable of eliminating *A. baumannii* and *P. aeurigonosa* quickly and efficiently, avoiding selective pressure and even generating better potential inhibition than conventional drugs. Although they are good alternatives because they act through interaction with microbial membranes, many AMPs have poor pharmacological profiles, and alternatives are being studied to overcome these barriers, like the chemical modification and synthesis of new peptides (Moradi et al., 2016).

The mechanism of action can be modified by targeting the bacterial membranes—producing differences in different cellular lipid compositions—or non-membrane, with intracellular targets (Preethi and Anbarasu, 2022). Thus, some modifications might result increasing antimicrobial potency, like disulfide bonds (Ravishankar et al., 2016), hydrophobicity (Chen et al., 2007), conformational freedom (Li et al., 2011) and N-Methylation (Montaser et al., 2011), but there are natural antimicrobial peptides with potential activity against Mtb infection (Preethi and Anbarasu, 2022). Accordingly, many AMPs could also eliminate cancer cells, their main objective being membrane receptors differentiated from those of healthy cells. ACPs can be classified into two large groups where their main differentiation is damage against healthy mammalian cells (Papo and Shai, 2005).

Studies on the antimicrobial effect of AMPs have also indicated a possible alternative when applied synergistically with some conventional drugs, sensitizing resistant bacteria or opening the way for the entry of obsolete drugs. Thus, a sequence M(LLKK)_2_M in combination with rifampicin was reported to act against resistant strains, and some strategies shown by the same authors indicated that the addition of cysteines in the sequence did not affect the activity, as compared with the addition of methionines (Khara et al., 2014).

The immunomodulatory capacity of AMPs is obviously one of the most favorable factors in the treatment of an infectious or immunosuppressive disease (Jin and Weinberg, 2019). AMPs can stimulate various molecules present in the immune system, such as chemokines, to respond to disease. Naturally, the production of AMPs occurs through the stimulation of abnormal factors such as inflammation, autoimmune diseases or co-infections (Magana et al., 2020). Previous studies have shown that the use of these peptides applied as oral additives or in localized infections was able to neutralize the disease or control the spread of pathogenic agents (Molchanova et al., 2017). The use of AMP Ctx(Ile^21^)-Ha had a great effect against resistant *Salmonella enteritidis* when pH-responsible coated microparticles were applied orally in chicks (Roque-Borda et al., 2021a) and pigs or wild-type peptide in beef cattle nutrition (Silveira et al., 2021). These studies revealed that the antimicrobial peptide was able to cross the intestinal barrier and eliminate pathogenic bacteria from systemic organs (liver > spleen > intestine; Roque-Borda et al., 2022b). In addition, depending on its microencapsulating characteristics, the peptide was capable of acting against many other intestinal infectious bacteria such as *Salmonella typhimurium*, *Salmonella enteritidis*, *Salmonella* Heidelberg, *Salmonella* Infantis (isolated from symptomatic chickens), and *Escherichia coli* (Roque-Borda et al., 2023).

The application of AMPs in the treatment of bacterial infections would imply their use against clinical isolates, multi-and extensively drug-resistant (M or XDR) strains such as GL13K, which had excellent activity against *Acinetobacter baumannii* MDR and XDR (Gorr et al., 2022), or Chex1-Arg20 hydrazide, which was also effective against the formation of biofilms of the same bacterial genus (Li et al., 2022). The formation of biofilms is another major problem when opportunistic bacteria try to restrict the passage of drugs and thus prevent their action against themselves, for which they produce various compounds based on proteins, polysaccharides and exogenous DNA; this process allows the biofilm to be more aggressive and in many cases untreatable (De Pontes et al., 2022). The alpha-helical structure and beta-sheet distribution of this peptide, as well as that of other peptides, would be related to its antimicrobial activity and its broad spectrum. This is the case of Chex1-Arg20 hydrazide rich in proline (De Pontes et al., 2022). An important group of natural AMPs belongs to the bacteriocins from the innate immunity of living beings; some bacterial communities produce them to protect themselves from other invading bacteria (Simons et al., 2020).

An interesting proposal for drug discovery and design is the use of bioinformatics tools, such as molecular docking, that predict the possible sequences with the best peptide-receptor interaction. Receptors are molecules that can be present in the bacterial membrane or in some specific component generated by its own resistance and are capable of being recognized by AMPs for possible degradation or elimination (Ali et al., 2017). Some studies have shown that molecular docking followed by an *in silico* ADMET study would help reduce costs and study time, since its prediction would facilitate the faster discarding of inactive molecules (Rampogu et al., 2018; Xiong et al., 2021). It was shown that this tool makes it possible to obtain promising sequences for various diseases and that many of these sequences can be found within macromolecules such as proteins or enzymes. Some enzymes produced by bacteriophages intensify the selective activity against MDR bacteria, and certain AMPs were found within these structures, which after their isolation and application demonstrated excellent activity (David et al., 1980). Thus, the lysine B fraction of bacteriophage D29 was a great source of AMPs when its composition was studied *in silico*, and it showed a high interaction with Mtb and cancer cell-specific receptors (Snyder et al., 2018; Dulberger et al., 2020). Table 2 shows the potential characteristics of antimicrobial peptides applied against strains of *M. tuberculosis* and potential candidates for new drugs.

**Table 2:** Antimicrobial bioactive peptides against *M. tuberculosis.*

AMP	MIC in *M. tuberculosis* (μM)	Cytotoxicity (μM)	Highlights	References
IP-1	32	HEK293T and MEF: 50	It induces autophagy of infected macrophages	Coyotl et al. (2020), De Pontes et al. (2022)
B1CTcu5	5.54	THP1: 6.62–662	It induces cavitation of the mycobacterial cell wall.	Abraham et al. (2020)
NZX	6.3	Monocytes: >100	It reduces the bacterial load within 5 days of treatment.	Mustafa et al. (2022)
S760	0.018	Macrophages: 100	Produced by lactic acid bacteria, it has immunomodulation action.	Sharma et al. (2019)
1PNB	*	-	It is a competitive inhibitor against glucose-1-phosphate thymidylyltransferase of *M. tuberculosis.*	Mustafa et al. (2022)
LL-37	1.11′	Eukaryotic cells: down to15	It resides in lysosomes and disrupts the mycobacterial cell wall	Deshpande et al. (2020)
Ub2	*	J774 A.1: 500	In addition to proteases and lipases in the lysosomal lumen, it can promote mycobacterial killing.	Alonso et al. (2007), Singh et al. (2019)
Hcl2	3.72	THP-1: 99.3	It is derived from the cytochrome c oxidase subunit 3 and disintegrates the mycobacterial cell wall.	Samuchiwal et al. (2014)
VpAmp1.0	17.4	Red blood cells: 9.2	This AMP is derived from the venom glands of the scorpion *Vaejovis punctatus.*	Ramírez-Carreto et al. (2015)
VpAmp1.1	5.4	Red blood cells: 33.7	It is a variant of VpAmp1.0, with better biological activities.	Ramírez-Carreto et al. (2015)
VpAmp2.0	21.4	Red blood cells: 167	It is a variant of VpAmp1.0, with better biological activities.	Ramírez-Carreto et al. (2015)
VpAmp2.1	13.6	Red blood cells: 103.5	It is a variant of a peptide derived from *Vaejovis punctatus.*	Ramírez-Carreto et al. (2015)
Buforin I	> 12,2	HSF: 331 e 6,620	It is derived from *Bufo bufo gargarizans*; it has antibacterial and antifungal activity.	Portell-Buj et al. (2019), Roshanak et al. (2021)
Mastoparan	43.3	Hemolytic activity: 331	It is derived from *Vespula lewisii*; it has antibacterial activity.	Portell-Buj et al. (2019), Rungsa et al. (2022)
Histatin 5	*	MMP-2 and MMP-9: 0.57 and 0.25	It is secreted by parotid and submandibular glands; it has antibacterial, antiviral and antifungal activity.	Gusman et al. (2001), Portell-Buj et al. (2019), Ikonomova et al. (2020)
Magainin I	*	HT29: 74.9	It is derived from *Xenopus laevis;* it has antibacterial and antiviral activity.	Portell-Buj et al. (2019), Hassan et al. (2021)
Magainin II	*	PBMC: 132	It is derived from *Xenopus laevis;* it has antibacterial, antiviral and antifungal activity.	Horváti et al. (2017), Portell-Buj et al. (2019)
Cecropin PI	*	PBMC: 1324	It is derived from *Ascaris suum;* it has antibacterial activity.	Han et al. (2011), Portell-Buj et al. (2019)
Cecropin A	> 44	Erythrocytes: 1118.78	It is derived *Hyalophora cecropia;* it has antibacterial and antiviral activity.	Wei et al. (2016), Portell-Buj et al. (2019)
Cecropin B	*	RAW264.7: > 25	It is derived *Antheraea pernyi;* it has antibacterial and antifungal activity.	Wang et al. (2018), Portell-Buj et al. (2019)
Melittin	> 45.7	PBMC: 0.941	It is derived from *Apis mellifera;* it has antibacterial, antiviral and antifungal activity.	Horváti et al. (2017), Portell-Buj et al. (2019)
HNP1	*	Kidney cells: 29	Synergistic activity with LL-37.	Kalita et al. (2004), Drab and Sugihara (2020)
Callyaerin A	2	THP-1 and MRC-5: <10	It is derived from *Callyspongia aerizusa*	Daletos et al. (2015)
Callyaerin B	5	THP-1 and MRC-5: <5	It is derived from *Callyspongia aerizusa*	Daletos et al. (2015)
Callyaerin C	40	THP-1 and MRC-5: <100	It is derived from *Callyspongia aerizusa*	Daletos et al. (2015)
Laterosporulin10	0.31–4.0	Blood cells >20 and mammalian cells >30	Bacteriocins from *Lactobacillus, Bacillus, Paenibacillus* and *Brevibacillus* sp.	Baindara et al. (2016)

* Values just in μg/mL without molar mass in the reference.

In addition to Tables 2, 3 lists recent examples of peptides with activity against other intracellular pathogens, showing that this field of study is wide and offers many candidates for new drugs.

**Table 3:** Some antimicrobial bioactive peptides against other intracellular pathogens.

AMP	Intracellular Pathogen	References
A11	*S. typhimurium*	Sengkhui et al. (2023)
AMP1	*E, coli, K. pneumoniae, A. junii, E. faecalis* and *P. aeruginosa*	Chaudhary et al. (2023)
AMP2	*K. pneumoniae, A. junii, E. faecalis* and *P. aeruginosa*	Chaudhary et al. (2023)
AMP3	*K. pneumoniae, A. junii, E. faecalis* and *P. aeruginosa*	Chaudhary et al. (2023)
AvBD	*Pseudomonas aeruginosa* and *Methicillin-resistant Staphylococcus pseudintermedius*	Yang et al. (2018)
LEAP2	*A. hydrophila, E. coli, P. aeruginosa, S. enterica*, and *S. aureus*	Chen et al. (2023)
P36	*P. aeruginosa*	Pandit et al. (2022)
PLNC8 αβ	*Staphylococcus* spp.	Wiman et al. (2023)

AMPs tend to have good antimicrobial activity, but many of them have low selectivity since they interact mainly with the ionic charges of the bacterial membrane compounds (Magana et al., 2020). However, it has been reported that their structural modification could generate better antimicrobial selectivity and decrease its cytotoxicity. Table 4 shows the factors for and against AMPs, since there are several parameters to be considered for a possible scaling strategy. AMPs tend to have multiple mechanisms of action, which allows them to be membranolytic or not at the same time, an effect that can occur when AMPs are dependent on concentration, size or structure (Zhang R. et al., 2020). Alternatively, unconventional amino acids have been used to generate mimetic peptides, where hybrid combinations or unusual amino acids are used, which will generate molecules such as peptoids (α and β), β-peptides, α-peptide/β-peptoids, α/Υ N-acylated N-aminoethyl peptides (AApeptides), oligoacyllysines (OAKs), among others (Molchanova et al., 2017).

**Table 4:** Strengths, weaknesses, opportunities, and threats (SWOT) analysis of peptides with therapeutic properties (Fosgerau and Hoffmann, 2015).

Strengths	Weaknesses	Opportunities	Threats
Good safety, tolerability and effectiveness. Potency and high selectivity. Predictability in metabolism. Less time to market. Decreased attrition rates. Standardized synthetic procedures.	Physically and chemically unstable. Susceptible to oxidation and hydrolysis. A propensity to aggregate. Fast elimination and short half-life. Generally not used for oral administration. Low permeability of the membrane.	Discovery of novel peptides, including fragmentation of proteins. Created sequences that are optimized and focused libraries. Building a formulation. In addition to parenteral, other modes of administration. Multipurpose peptides and conjugates.	Immunogenicity. New developments in proteomics, genomics and customized medicine. Many patents are about to expire. Cost and reimbursement conditions. Growing need for new medications in terms of safety and effectiveness.
Physicochemical instability and aggregation can be overcome using nanocarriers and/or nanoprotectants (Roque-Borda et al., 2022b). The oral application of AMPs can be potentiated using encapsulations or changing the L amino acids to unusual and D-amino acids, since several of them have been reported to tolerate the presence of proteases (Lu et al., 2020). The cell permeability of AMPs can be modified by the inclusion of fatty acids or cell-penetrating sequences (Zhang J. et al., 2020). It has been shown that several AMPs would not be mutagenic, which paves the way for new peptide-based drugs. The AMES test is usually used and corroborated with the comet assay (Hajisharifi et al., 2014).			

Due to the large number of reports on antimicrobial resistance, induced by selective pressure, crossed or naturally acquired, a possible return to the pre-antibiotic era is anticipated. This would also indicate a possible reaction in response to conventional cancer treatment and a higher rate of deaths caused by opportunistic infections. In this review, we focus on an important reason for the use of multifunctional peptides or peptides with double activity and no harmful effects on humans, such as Epinecidin-1 and its analogs. These peptides had excellent antibacterial activity against various pathogens and an anticancer effect (Neshani et al., 2019). Some articles have reported the potential of AMPs and ACPs, showing that many of them were initially AMPs and were transformed into ACPs through the use of analogs (Table 5).

**Table 5:** Anti-inflammatory bioactive peptides.

Peptide	LC cell lines	Cytotoxicity (IC_50_)	Type/Structure	Highlights	References
Smp24	A549, H3122, PC-9, and H460: 4.06 to 7.07 μM	MRC-5: 14.68 μM	AMP	The peptide was able to kill A549 cells through the cell membrane, mitochondrial and nuclear destruction.	Nagaraj et al. (2022)
P-113 and its derivatives	H1975, A549 and PC 9 21.32 to >200 μM	NR	AMP	AMPs induce the death of immunogenic cells derived from cancer lines and release potent danger-associated molecular patterns.	Cheah et al. (2022)
NKTP-3	A427	NR	Cell-permeable Cyclic D-peptide	Dual-targeting (NRP1 and KRASG12D) peptide for therapy of LC.	Zhou et al. (2022)
BR2-2xPPD	A549, PC9-6 M, PC9-GR and PC9-ER (resistant cell lines): 2.5 μM	BEAS-2B and HaCaT: non-toxic	Cell-penetrating peptides (CPP)	CPPs induced cell cycle arrest by inhibiting the expression of cyclin D1 and CDK2 genes in A549 wild-type epidermal growth factor receptor cells.	Kaewjanthong et al. (2022)
P7	A549 and H1975, A549/CD133+ cells: 50 ηM.	NR	Peptide-drug conjugate	Docetaxel-P7 induced unfolded protein response and subsequent apoptosis by degrading Hsp90, while awakening and killing the dormant cancer stem cells.	Jiang Y. et al. (2022)
D-LAK-120A	A549, H358, H1975, and HCC827: 4 to 5.5 μM	Healthy lung cell line: 8.40 μM	AMP	D-LAK-120A inhibited cancer cell proliferation *via* both membranolytic and non-membranolytic pathways. AMP significantly inhibited colony formation and cancer metastasis *in vitro*.	Patil and Kunda (2022a)
EIP103	A549: 1 μM and H446: 10 μM.	DC2.4: non-toxic	Targeting peptide + CPP	Peptides that can specifically target nucleus receptor LC and improve the anti-tumor efficacy.	Jiang M. et al. (2022)
Mastoparan	A549: 1.3 μM	NR	Peptide-drug conjugate	Significantly higher cell counts were found in G2-M phase after treatment with mastoparan-alendronate sodium nanoconjugates.	Alhakamy et al. (2022)
ACPP-p21Ras scFv	A549, SW480, U251 and Huh7	BEAS-2B	CPP	Potential antitumor drug for Ras gene-driven LC	Du et al. (2022)
Peptide from circPPP1R12A-73aa protein	A549 and H1299	BEAS-2B	NR	NSCLC cell proliferation was promoted by circPPP1R12A-73aa translated from circPPP1R12A through the AKT pathway.	Zhao et al. (2022)
d-peptide VAP	A549-luc	NR	Conjugate peptides	The D peptide modification markedly enhanced the tumor-targeting efficiency of nanodisks, thereby improving the anti-tumor properties of non-small cell LC efficacy of the drug delivery system.	Song et al. (2022)
CIGB-552 peptide	NCI-H460, A549 and TC-1 (*in vivo*)	NR	Antitumoral peptide	The results demonstrated a clear synergic effect between 37.5 μM of CIGB-552 and 5 μM of cisplatin under a concomitant scheme, on proliferation inhibition, cell cycle arrest, apoptosis induction and oxidative stress response.	Gomez Rodriguez et al. (2022)
CP7	A549: 14.94 μg/mL	NR	Targeting peptide	*In vivo* experiments proved that siRNA/liposome-PEG-CP7 has excellent tumor targeting and tumor inhibition function in tumor-bearing mice.	Dong et al. (2022)
Nisin ZP	A549: 132.4 μM H1299: 137.3 μM	HEK293: > 300 μM	AMP	The cell cycle arrest suggested accumulation of cells in initial G0/G1 phase, which ultimately culminated in apoptotic cell death of NSCLC cells regardless of p53 tumor protein expression.	Patil and Kunda (2022b)
cRGD polypeptide	LL/2	NR	Conjugate peptides	It exhibited good tumor-targeting capability, good biodegradability and biocompatibility. Combined treatment displayed enhanced anti-tumor and anti-metastatic ability in LC therapy.	Zhang et al. (2022)
Laterosporulin10 (LS10)	MCF-7, HEK293T, HT1080, HeLa and H1299: <10 μM	Prostate epithelium cells (RWPE-1): >15 μM	AMP	Laterosporulin10 is an anticancer bacteriocin that, at low and high doses, induces the death of cancer cells by apoptosis and necrosis, respectively. In light of the study’s overall findings, AMP is an anticancer peptide that may be further developed for medicinal uses.	Baindara et al. (2017)

Some mechanisms of action based on the internalization of AMPs that are not directly related to the disruption of the membrane in cells are classified as (i) inhibitors of biosynthesis and metabolism of nucleic acids, (ii) inhibitors of biosynthesis and of protein metabolism, (iii) inhibitors of protein folding, (iv) protease inhibitors, (v) inhibitors of cell division, (vi) inhibitors of cell wall biosynthesis and (vii) binding peptides of lipopolysaccharide (Le et al., 2017). Histatins, Bactencin 7, and Apidaecin are examples that show that lipids or fatty acids can help with the translocation of AMPs within the cell membrane, which will depend on their beta or alpha-helix structure; mediated by stereospecific receptors, AMP can be located in the cytoplasm to exert their antimicrobial action (Le et al., 2017). However, this effect can limit the action of AMPs since their broad-spectrum activity can generate some type of cytotoxic or immunological response. Specifically in Mtb, the most widely used and promising strategies are focused on the search for new inducers of autophagy (Arranz-Trullén et al., 2017). Some prominent AMPs in cell permeability with intramacrophage activity against Mtb are those derived from human neutrophils, such as 1, 2, 3 (hNP1-3), lipocalin-2 and human cathelicidin, which are usually expressed when Mtb begins its extracellular multiplication (Gutsmann, 2016). These AMPs agglomerate in the bacterial cell wall, producing lesions through the formation of warts, but they also enter the cytoplasm, neutralizing bacterial DNA synthesis. Likewise, in recent years, have been reported on the multiple mechanisms of action against intramacrophagic pathogens, even Mtb with defensins, cathelicidins, hepcidin, NK-lysin, granulysin and ubiquitin, among others, demonstrating that AMPs can be selective and effective against resistant strains (Schlusselhuber et al., 2013; Baindara et al., 2016; Moussouni et al., 2019; Abraham et al., 2020; De Singulani et al., 2021).

Defensins play an important role in the innate immunology of the lung because the production of these AMPs has been reported to increase when there is colonization of *Mycobacterium* (Gutsmann, 2016). For example, the human β-defensins (hBD 1 or 2) can cross macrophages and phagosomes and inhibit bacterial growth. It has even been shown that their application combined with vitamin D significantly increased the inhibition percentage (Nickel et al., 2012); in the same way, prevention or prophylactic effect was achieved by combining defensins with L-isoleucine (Rivas-Santiago et al., 2015). However, it has already been demonstrated that hBD 2 can induce the proliferation of lung cancer cells through ABCG2 in a dose-and time-dependent manner (Gao et al., 2016; Ghosh et al., 2019). Laterosporulin10 is an AMP isolated from *Brevibacillus* sp. that has been shown to have excellent cellular permeation properties in macrophages and activity against Mtb (Baindara et al., 2016), but which at the same time induces cell apoptosis in cancer cells (HeLa cells; Baindara et al., 2017).

Some selectivity strategies have generated positive effects in relation to the hemolytic activity of the AMP and the inclusion of fatty acids is a double-edged sword since these substances can improve their antimicrobial activity in the chain but, in turn, increase their cytotoxicity or hemolytic activity. Some authors have reported that fatty acids between 8 and 10C may be more active and selective against resistant bacteria, as well as less toxic (Zhong et al., 2020). Nevertheless, these factors must be carefully studied as they depend on the hydrophobicity of the AMP and the total load that is handled. It was previously reported that the hemolytic activity and the hydrophobicity are easily predictable when the HPLC profiles are evaluated. They may be predicted by using the retention time and the % of acetonitrile, which may help to analyze the potential of different AMPs (Frederiksen et al., 2021). One of the advantages of including fatty acids is the ease of permeation of molecules within human cells. For this reason, this strategy can be initially generated with *in silico* molecular prediction studies with different virtual platforms. The first step would be to narrow down and select the best candidates with antimicrobial or anti-Mtb activity, as described in https://webs.iiitd.edu.in/raghava/antitbpred/ (Usmani et al., 2018), then analyze their hemolytic activity (Salem et al., 2022) and finally perform molecular docking with cell membrane proteins (Roque-Borda et al., 2023).

Another alternative would be the inclusion of cell-penetrating peptides (CPPs), making them hybrid and trying not to alter the net charge and hydrophobicity. CPPs can enter human and/or animal cells to interact with molecules internally, so many AMPs may also have permeation capacity, being able to be AMP or CPP at the same time (Xie et al., 2020). An example of hybridization was reported for Iztli Peptide 1, designed using a fraction of the *Saccharomyces cerevisiae* α pheromone sequence (cationic AMP) and a short CPP (without antimicrobial activity). This study showed that the hybrid AMP was capable of penetrating cells regardless of endocytosis, which paved the way for the design of new multiple-action molecules with improved cellular compatibility (Rodriguez Plaza et al., 2014). It was previously reported that the inclusion of arginines or their replacement with tryptophans could modify the behavior of AMPs by converting them into CPPs while maintaining their antimicrobial activity and their target cell selectivity (Kardani et al., 2019).

Nanotechnology as a drug delivery tool has taken on great importance during the application and transport of promising AMPs against intracellular bacteria, since many times their size and biocompatibility permeate cell membranes or easily cross them without generating an immediate immune response (Roque-Borda et al., 2022a). Nanoparticles (NP) are promising agents mostly for intramacrophage or phagosome antimicrobial action, since their construction implies the design of specific targets for their controlled release, so that large but not excessive drug concentrations can be administered (Gharatape et al., 2016). Some previous studies reported the use of polymers modified on their surface with biomacromolecules or antibodies for the recognition of proteins expressed only by tumor cells, or release conditions in slightly acidic environments that are characteristic of cancer cells (Richards et al., 2017; Marques et al., 2020). NPs can also enter infected macrophages and release AMPs to exert their action, especially when they do not have cell-penetrating characteristics (Tenland et al., 2019; Meng et al., 2023).

## 2. Discussion

Lungs have historically been considered sterile in health and were initially omitted from the list of priority organ systems in the Human Microbiome Project (HMP), but subsequent studies by metagenomic analysis have since demonstrated that the lower respiratory tract is replete with diverse communities of bacteria both in health and disease (Dickson et al., 2013; Liu et al., 2020) containing about 10 to 100 bacteria per 1,000 human cells (Sze et al., 2012). Due to the importance of inflammation and cancer, the presence of microorganisms can be related to the initiation and/or progression of cancer. Some microorganisms have already been detected in patients with lung cancer. Most microorganisms colonize the tumor microenvironment, which can induce the production of inflammatory molecules (Molina-Romero et al., 2019). As shown in Table 1, some gut microbiota-related microorganisms are present in lung cancer patients.

The respiratory tract and gut can communicate with each other *via* some anatomical processes such as micro-aspiration and inhalation (Zhao et al., 2021). Other studies have pointed to the existence of a gut-lung axis (GLA) that can interfere with the immune responses and change the course of respiratory diseases (Enaud et al., 2020). For example, the severity of Mtb infection may be correlated with gut microbiota (Namasivayam et al., 2018). Still, some AMPs can be delivered to the gut by means of living therapies previously designed using probiotic bacteria (Geldart et al., 2016). These therapies are advantageous because probiotics help to maintain the host’s health and they are a novel approach to lung-directed treatment against TB infection (Gareau et al., 2010; Mejía-Pitta et al., 2021). Gut-related microorganisms in lung cancer can be classified into two types of relationship, namely their presence in the tumor microenvironment or a dysbiosis in the gut microbiome. Some microorganisms such as *Bifidobacterium* have been reported to be potential biomarkers of lung cancer, due to their decline in the gut microbiota (Zhuang et al., 2019; Zhao et al., 2021). These characteristics of the gut microbiota in lung cancer patients may affect therapy effectiveness and prognosis.

The relationship between TB and LC is the subject of much debate. The chronic inflammation caused by the colonization of active Mtb can be one of the mechanisms increasing the probability of developing this carcinoma. Chronic inflammation caused by this infection has been linked to cell dysplasia, as well as squamous LC (Broussard et al., 2009; Skowroński et al., 2015). LC can be promoted by persistent local inflammation, which contributes to damage of the pulmonary epithelium, and the cytokines released can induce the proliferation of lung epithelial cells (Ballaz and Mulshine, 2003). The main reason for describing this relationship is the development of carcinoma from TB scars (Brenner et al., 2011; Cukic, 2017). This relationship may be based on some mechanisms and interactions. For example, TB-related chronic inflammation can induce genetic mutations (Vento and Lanzafame, 2011). A close association between metastatic squamous cell carcinoma (SCC) and TB lesions has been demonstrated (Nalbandian et al., 2009). Lung parenchyma is involved in both TB and LC (Bhatt et al., 2012). The production of inflammatory cytokines can be related to cancer development (Cicėnas and Vencevičius, 2007; Engels et al., 2009). The development of the granulomas in an Mtb infection may generate a microenvironment predisposed to malignant transformation. This microenvironment may lead to changes in the epithelium of the lung caverns, calcified lymph nodes and old scars in the bronchi (Cukic, 2017). Still, the pneumocytes are infected and activated, with the induction of autophagy, secretion of pro-inflammatory mediators and cell death as outcomes (Rodrigues et al., 2020). Polverino et al. (2016) demonstrated that ligands of proliferation are more highly expressed in patients with both chronic inflammation and non-small lung cancer cells than in patients with only chronic inflammation or cancer. The expression of these ligands can be related to the progression of both, inflammation and cancer (Polverino et al., 2016).

It is important to note that cancer and TB can mimic each other at clinical, biological and radiological levels. This can be hazardous for the patient. A patient with LC but diagnosed with TB will receive chemotherapy to no effect. In fact, this therapy would deteriorate the patient’s response. On the other hand, receiving treatment for TB when the patient has cancer could lead to a complication and eventual death of the patient. Even in a concomitant condition, when a patient is correctly diagnosed with LC but also has TB, the healing process can be complicated (Malik et al., 2022).

**Figure 2:**
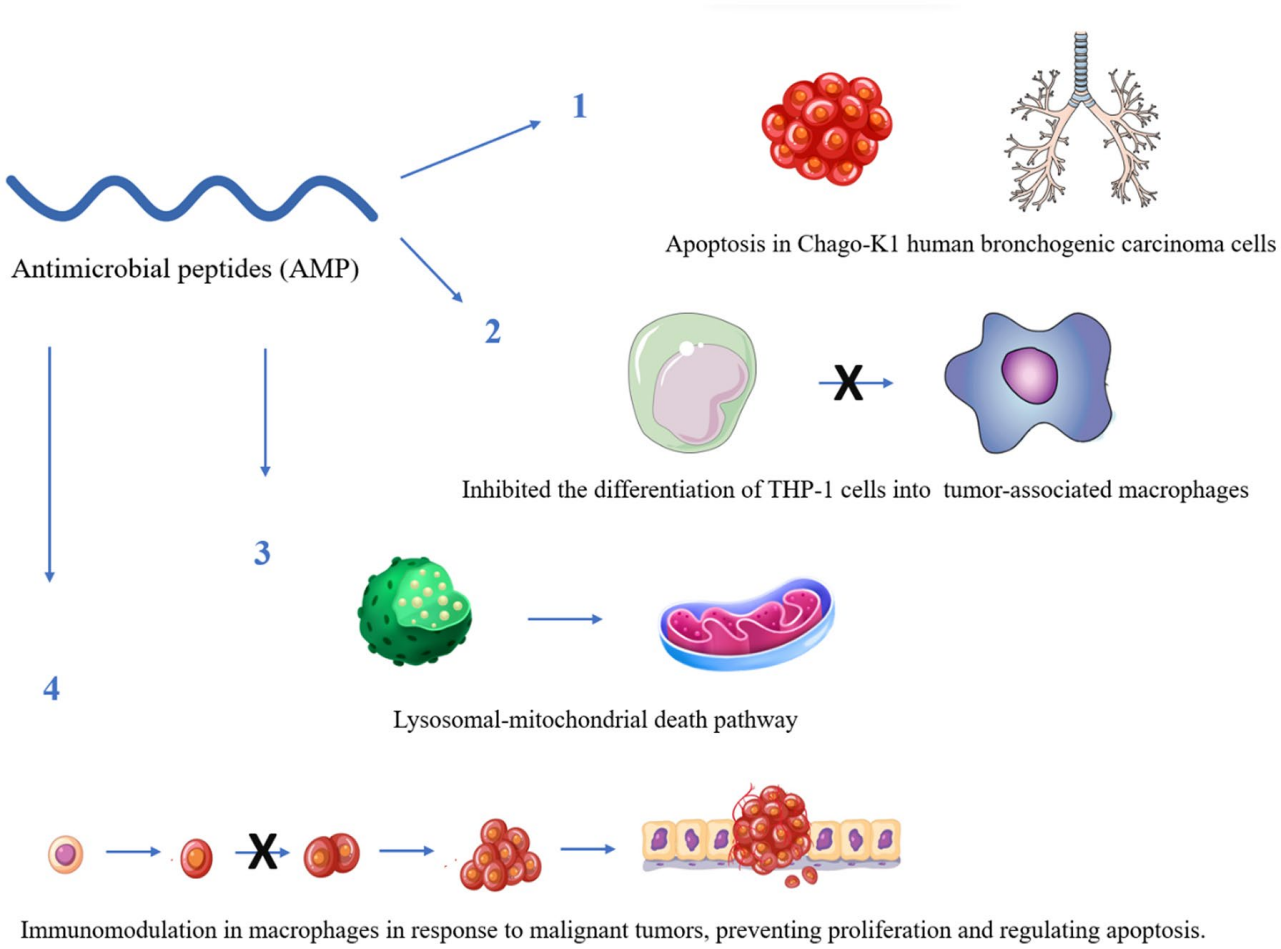
Some anticancer mechanisms of antimicrobial peptides with known activity against *Mycobacterium tuberculosis*. The figure was created by Biorender.com, partly generated using Servier Medical Art, provided by Servier, licensed under a Creative Commons Attribution 3.0 unported license.

Some reports have indicated that AMPs would be able to recognize different cancer cell receptors for efficient molecular targeting (Figure 2). Therefore, they could be a relevant alternative to the use of AMPs, for application against cancer and could be a relevant alternative against bacteria (Zhang et al., 2019).

Kunda (2020) compiled different applications of these peptides against an unchained proliferation of non-small cell LC, because AMPs can develop similar mechanisms of action against bacteria; these peptides may interact with negatively charged molecules of cancer cells such as phosphatidylserines, negative glycoproteins and glycosaminoglycans, i.e., highly expressed in the presence of oncocells. One of the main problems with the use of AMPs in the treatment of cancer is their cellular non-specificity, which could damage healthy cells at the same time (Hilchie et al., 2019). However, there are already some strategies to improve the peptide sequence by obtaining specific target analogs that could generate better action and fewer side effects (Yang et al., 2022). Table 6 shows the main AMPs that have been relevant in the last three years, with potential anti-Mtb and anticancer activity. The results show that few studies have proven the activity for both diseases, but it is possible to obtain drugs with double function and activity.

**Table 6:** AMPs with proven anticancer activity and activity against *Mycobacterium tuberculosis*.

AMPs	Potential anti-cancer/Mtb activity	References
Melittin	Melittin is an AMP with relevant characteristics that have been very useful against several cancer cell lines, and it is currently advancing in the clinical phase. Importantly, it induced G1 cell cycle arrest and apoptosis in Chago-K1 human bronchogenic carcinoma cells and inhibited the differentiation of THP-1 cells into tumor-associated macrophages; also *via* inhibition of miR-183, it induced NSCLC apoptosis. Previous studies in clinical isolates of Mtb revealed that it has a slight bactericidal activity.	Tipgomut et al. (2018), Portell-Buj et al. (2019)
Cecropins	Cecropins are an important family of peptides, many of them found in insects. They were first isolated from *Hyalophora cecropia*. Some peptides derived from *Dichotomius satanas* and *Onthophagus curvicornis* dung beetles exhibited anti-Mtb activity but Satanin-1 also had *in vitro* antitumor activity against THP1 cells, indicating that it may be used as an anticancer agent. Some authors described its effective activity against bladder cancer, and it did not show cytotoxicity in murine fibroblasts.	Suttmann et al. (2008); Henao Arias et al. (2021)
Cathelicidins	Cathelicidins are a large group of AMPs, many of which are produced by humans. A study reported that modulating the expression of these peptides would be efficient during treatment against MDR Mtb. The authors recommended some exogenous dehydroepiandrosterone, phenylbutyrate, or histone deacetylase inhibitors, which promote the production of cathelicidins and human beta-defensins 2 and 3. Another clinical study performed in Zambia indicated the use of vitamin D in combination with LL-37 for preventive use. Likewise, some LL-37 analogs were able to kill A459 cancer cells from human lung adenocarcinoma. Some studies have shown the potential of LL-37 in immunomodulation in macrophages in response to malignant tumors, preventing proliferation and regulating apoptosis. These results show that AMPs have potential anticancer activity for possible clinical uses and feasible development of a new drug for preventive or effective treatment.	Chen et al. (2018), Tzitzilis et al. (2020), Marin-Luevano et al. (2021), Rao Muvva et al. (2021), Rodríguez-Carlos et al. (2021), Lungu et al. (2022)
Brevinins	Brevinins are AMPs isolated from frogs from different parts of the world. B1CTcu5 from the skin secretion of *Clinotarsus curtipes* was found to be non-toxic and to eliminate intracellular Mtb. These results have led to the option of using the cyclic forms of AMPs by inducing the formation of disulfide bridges, thus increasing their activity; this strategy can be considered favorable under oxidizing conditions. At the same time, Brevinin-2R1 showed a semi-selective activity that kills cancer cells by a mechanism involving the lysosomal-mitochondrial death pathway, important data for the development of new drugs and the discovery of new treatment pathways.	Ghavami et al. (2008), Abraham et al. (2020)

Derived by the endogenous cationic peptide Brevinin-1 family, the B1CTcu5, of amphibian origin, was able to inhibit Mtb in culture, as well as intracellular conditions, by permeabilizing the thick cell wall of Mtb to impart bactericidal activity without causing any damage to the macrophages. B1CTcu5 can mimic the environment by adopting an alpha-helical conformation in the membrane. This characteristic contributes to the activity against prototype Gram-negative and Gram-positive bacteria. The hydrophobic interaction between amphipathic AMP and the cell membrane makes a specific peptide–lipid complex, which may produce alterations in the bacterial membrane, such as thinning, pore formation, altered curvature and localized perturbations (Abraham et al., 2020).

A recent example is LL-37, an endogenous AMP derived from Cathelicidin. It is also an endogenous cationic peptide expressed in human immune cells, with activity against extra and intracellular Mtb, mainly through the disruption of the bacterial cell wall upon binding, causing disintegration and rapid rupture within minutes. Moreover, other results the same group showed that LL-37 is internalized by macrophages and localized within the membrane of early endosomes labeled with antigen 1 protein (Deshpande et al., 2020). Other findings suggested that by “nonclassical” mechanisms, vitamin D contributes to protection against TB and can act in combination with LL-37 (Martineau et al., 2007). For lung cancer, studies have shown that LL-37 increased tumorigenicity and significantly larger tumor mass in human lung cancer cells (von Haussen et al., 2008), but analogs have significantly improved cytotoxicity against cancer cell line A549 cells (Tzitzilis et al., 2020).

Finally, in addition to the activities mentioned in Table 6, a recent study suggests a combination of amphipathic α-helical N-terminal region of cecropin A and hydrophobic N-terminal of the bee venom melittin, known as CP26. This combination is a 26-amino acid long β-helical peptide with activity against both susceptible (H37Rv) and MDR strains of Mtb (Rivas-Santiago et al., 2013), but this combination of peptides has not yet been studied against lung cancer.

The antimicrobial peptides can also be designed to combat bacteria and cancer cells through re-engineering into anticancer peptides. Both bacteria and cancer cells possess an electronegative surface that the peptides can break because they are cationic amphiphiles. Aronson et al. (2020) tested this hypothesis with an AMP originally designed to kill *M. tuberculosis* and found a powerful result against ovarian cancer, opening new paths to study alternatives that are also potent against lung cancer (Aronson et al., 2020).

## 3. Conclusion

The most debated hypothesis is that an Mtb infection can trigger such a complex inflammatory environment in the lung that it unintentionally induces normal cells to differentiate into cancer cells, initiating lung cancer. Many researchers are studying this link, but more experimental data are needed to establish a clear mechanism between the diseases, especially through the study of clinical-human samples. A few other researchers suggest the opposite, namely that there is a predisposition to Mtb infection precisely because of a depressed immune system and tissue affected by the growth of cancer cells, but these studies have not found an accurate mechanism proving this relationship.

Regardless of the disease that initially affected the patient, diagnostic methods for differentiating them are extremely necessary because they have similar symptoms, confusing and/or masking the secondary disease, which, in turn, can go untreated or treated incorrectly, thus worsening the clinical scenario. In addition, there is a need for new treatment alternatives that combat both diseases developed in the same organ, so that there is no unnecessary overlapping of drugs, causing greater toxicity or even antagonistic effects.

We report on four alternatives of antimicrobial peptides with current relevance, which were able to show activity against the mycobacteria as well as against lung cancer. Examples of peptides from the same class with action on TB and LC have been presented. However, no derivative or analog has yet been found that manages to act on both diseases for concomitant treatment, which opens up a field of study with infinite possibilities for alterations and combinations. These alternatives need more investment and further studies to prove the dual activity and develop dual-function drugs that minimize the abandonment of therapy when pulmonary diseases coexist.

## Author contributions

GP: bibliographic survey, bibliography analysis, discussion, and manuscript elaboration. LP, MR, FD, and PB: bibliographic survey and manuscript elaboration. CR-B and FP: supervision, idea, bibliography analysis, discussion, and manuscript elaboration. All authors contributed to the article and approved the submitted version.

## Funding

This study was supported by the São Paulo Research Foundation (FAPESP): Research Grant: 2020/13497-4, 2022/09728-6, 2021/14603-5 and 2020/16573-3. National Council for Scientific and Technological Development (CNPq): Productivity Research Fellows (PQ CNPq): 305408/2022-4. This study was financed in part by the Coordenação de Aperfeiçoamento de Pessoal de Nível Superior - Brasil (CAPES) - Finance Code 001.

## Conflict of interest

The authors declare that the research was conducted in the absence of any commercial or financial relationships that could be construed as a potential conflict of interest.

## Publisher’s note

All claims expressed in this article are solely those of the authors and do not necessarily represent those of their affiliated organizations, or those of the publisher, the editors and the reviewers. Any product that may be evaluated in this article, or claim that may be made by its manufacturer, is not guaranteed or endorsed by the publisher.
